# Comparison of in-and outpatients protocols for providence night time only bracing in AIS patients – compliance and satisfaction

**DOI:** 10.1186/1748-7161-8-6

**Published:** 2013-04-12

**Authors:** Zaid TJ Al-Aubaidi, Hans Tropp, Niels W Pedersen, Stig M Jespersen

**Affiliations:** 1 Odense university hospital, Odense, Denmark; 2 Linkoping hospital, Linkoping, Sweden; 3 Orthopaedic Surgical department, Odense university hospital/ Denmark, Odense, Denmark; 4 Department of Orthopedics, Odense University Hospital, Soender Boulevard 29, Odense C, DK-5000, Denmark

## Abstract

**Background:**

Skeletally immature patients diagnosed with adolescent idiopathic scoliosis (AIS) and a Cobb angle above 25degrees is usually treated with a brace. Standard protocols in many centers include hospitalisation for a few days for the purpose of brace adaptation and fitting. The aim of this study is to compare compliance and satisfaction in hospitalization and out patient clinic protocols, at the initiation phase of brace treatment.

**Materials and methods:**

Twenty-four consecutive patients with AIS were initiated with the Providence night time only brace at our department between October 2008 and September 2009. The first twelve patients were admitted for a maximum of 3 days during the initiation phase of brace treatment. The following twelve patients were initiated in an outpatient clinic set-up. In this later group, patients and parents were informed about the possibility to be admitted to the hospital, at the initiation phase but all patients chose to be treated as out patient’s protocol. All patients were evaluated by means of conventional x-ray and patients reported outcome measurements. The mean follow up was 6 months for the outpatient group (3-8) and 12 months for the hospitalisation group (9-14). Scoliosis Quality of Life Index (SQLI) was used together with the Odense Scoliosis questionnaire, which was developed for this study. Compliance was measured using the patients’ own statements and the Landauer compliance scoring system.

**Findings/results:**

The two groups’ matches regarding the age, Risser grad, Cobb angle and primary correction. There were no statistically significant differences between the two groups regarding the SQLI and the Odense Scoliosis questionnaire. The compliance was higher in the ambulatory group.

**Conclusion:**

Outpatient initiation of bracing in scoliosis seems to give the same correction but better compliance compared to initiation during hospitalization.

## Introduction

Scoliosis is a fixed lateral curvature of the spine of at least 10° measured by Cobb’s method [1-5]. Depending on the aetiology, scoliosis can be classified into various types with Idiopathic Scoliosis (IS) being the largest group. Despite much clinical, epidemiological, and basic science research, the aetiopathogenesis of IS remains unknown [1,4,5]. The largest subtype of this group is Adolescent Idiopathic Scoliosis (AIS), which by definition occurs before skeletal maturity in 3% of at-risk population, mostly girls between 10 and 16 years old [1,4-8]. Scoliosis can eventually result in severe curves of the spine. Thoracic curves above 80° Cobb angle can result in decreased pulmonary capacity, back pain, and significant cosmetic deformity [1,8]. The current treatment protocols for mild curves below 25° is observation alone or with physiotherapy, for curves equal or greater than 25° orthotic treatment is used alone or in conjunction with physiotherapy, while surgery is indicated for curves greater than 45° [1,4,5,7,9-11]. The use of bracing treatment for AIS continues to be controversial, but it is the only alternative treatment available that might prevent surgery for progressive curves in skeletally immature patients.

In many centers the initiation of brace treatment and brace fitting is performed under admission of the patient for few days. The objectives of this study are to evaluate whether an outpatient initiation could be done without negative impact on the treatment in the initiation phase and compliance to the use of the brace.

## Materials and methods

After approval of our institutional board of research, a prospective study was conducted and twenty-four consecutive patients diagnosed with progressive AIS were enrolled in the study. All the patients were initiated for brace treatment at our institution from October 2008 to September 2009. Inclusion criteria were skeletally immature patients (Risser grade 0-3) diagnosed with AIS with a Cobb angle between 25° and 40° and a curve apex at T8 or below. All patients received detailed written information regarding the disease and treatment. Exclusion criteria were all non-idiopathic scoliosis, patients with Risser grade 4-5 and patients refusing brace treatment. Radiological evaluation was done using the Sectra PACS electronic system, which has its own Cobb angle measurement tool. Consecutively the first 12 patients had their treatment initiated during hospitalization for three days. Thereafter, we changed the protocol and the following 12 consecutive patients had their treatment started as an outpatient procedure. The first group will be referred to as Hp group and the second as Op group, for the purpose of simplicity. Patients and parents were informed about the possibility of initiating brace treatment under hospitalization, but all patients chose to be treated as out patients. Follow-up was performed after a minimum of 3 months. The Scoliosis Quality of Life Index (SQLI) questionnaire was used [12]. This was translated to Danish, and then translated back to English by two independent medical professionals. The results of translation was checked by the corresponding author and found compatible with the original version. For the purpose of having a more applicable questionnaire to this study, Odense Scoliosis Questionnaire (OUH) was designed (Table 1). This questionnaire is based on three concepts, in which each has dimensional questions. Each question can be rated from 1; being the worst response to 5; being the best response. Compliance was measured using the patients’ own statements and the Landauer Compliance Scoring System [13]. This is a simple scoring system, depending on the subjective statement of the compliance, attendance to follow up, interruption of the treatment, skin reaction from brace wearing and signs of brace usage on the brace. Each item gives one point and in this way the highest compliance scoring being 5 and the lowest being 0.

### Procedure

Patients with a Cobb angle above 25° were recommended to use a Providence night time only brace [14]. The X-rays and prescription were sent to the same orthotist. Brace measures were taken using the measurement table and sent to Spinal Technologies (West Yarmouth, MA USA).The patients tried the brace on and any adjustment was made immediately. The orthotist instructed the patients and parents in how to put on the brace and markers were made to determine the amount of tension on the straps. The patients were asked to wear the brace for a minimum of eight hours, as the minimum recommended dosage [14]. The Hp patients were admitted for 2-3 days. The first day they were asked to put on the brace for one hour, then a couple of hours. If no problems where encountered, the patients could sleep with the brace on. Any probable adjustments were made immediately. The Op group was seen at the outpatient clinic two weeks after the brace was delivered. By this time, the patients were accustomed to sleep with the brace on. The Op group was seen by the corresponding author at the outpatient clinic. The brace was controlled, and tension was applied to the straps as marked by the orthotist. The patients had to lay supine for at least 20 minutes before X-ray was obtained. The primary correction was measured and registered. The patients were instructed by the treating doctor and a nurse about the importance of treatment. Both groups were seen after 4 months for a clinical and radiological control.

**Table 1:** Shows the Odense University Questionnaire

**Concept**	**Dimensions**	**No. of questions**
**Assurance and information**	Confidence to start brace treatment home	5
	Information level by the treating physician	
	Information level by the nurse	
	Information level by orthotist Information level by physiotherapist	
**Easiness of contact**	To the treating physician	3
	To the physiotherapist	
	To the orthotist	
**Preference for treatment (Home/in hospital)**	Preference to initiate treatment home	7
	Stress level to initiate treatment under admission	
	Stress level to initiate treatment under admission for other patients	
	Acceptance of treatment initiation at home	
	Practical issues to initiate treatment	
	Acceptance of treatment by other patients	
	Recommendation for start of treatment	

### Statistical analysis

The GraphPad Software (Avenida de la Playa La Jolla, CA 92037 USA) was used. Statistical comparisons were made using unpaired student *t-*test. The sample size calculation was made using a confidence level of 95% with a margin error of 28%.

## Results

**Figure 1:**
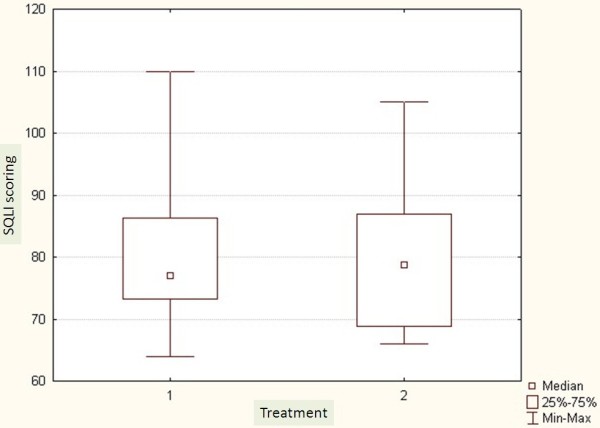
Scoliosis Quality of Life Index (SQLI). 1: Outpatient group and 2: hospitalized group.

**Figure 2:**
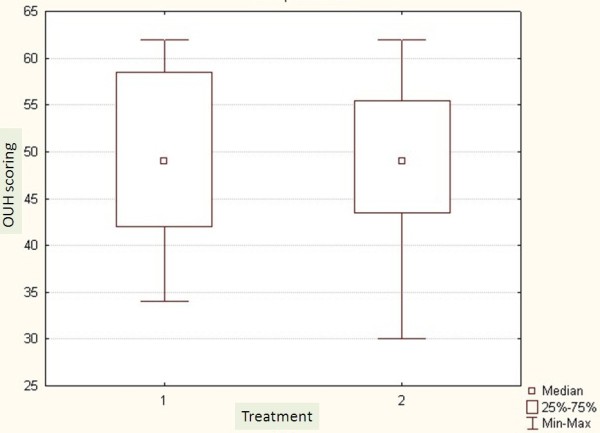
Odense University (OUH) Questionnaire. 1: Outpatient group and 2: hospitalized group.

**Figure 3:**
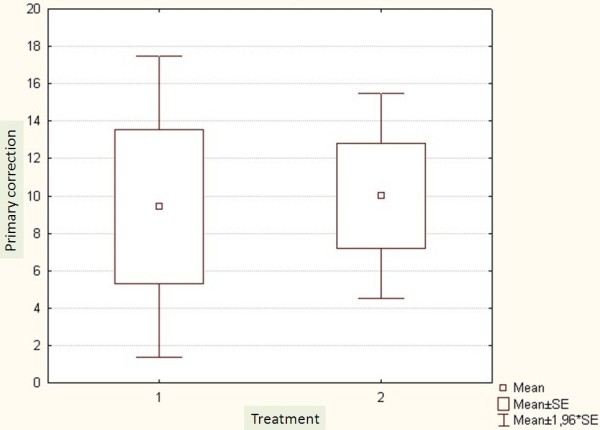
Primary corrections in both groups. 1: Outpatient group and 2: hospitalized group.

The average Cobb angle was 36^0^ ±8.6 in the Op group and 34^0^ ±9.9 in the Hp group. All patients were premenarchal and skeletally immature with a Risser grade ranging from 0 to 3. In the Op group the mean of Risser grad was 1.5 ± 1.3 and in the Hp group it was 1.3 ± 1.2. There were 24 females distributed equally in the two groups. Demographics are shown in Table 2. Mean age was 13 ±1.7 for the Op group and 12.5 ±2.4 for the Hp group. The mean follow up was six months for the Op (3-8) and twelve months for the Hp (9-14months). There was no significant difference concerning the SQLI or Odense University Questionnaire between the two groups (Figures 1, 2). There was no significant difference regarding the primary correction (Figure 3).

**Figure 4:**
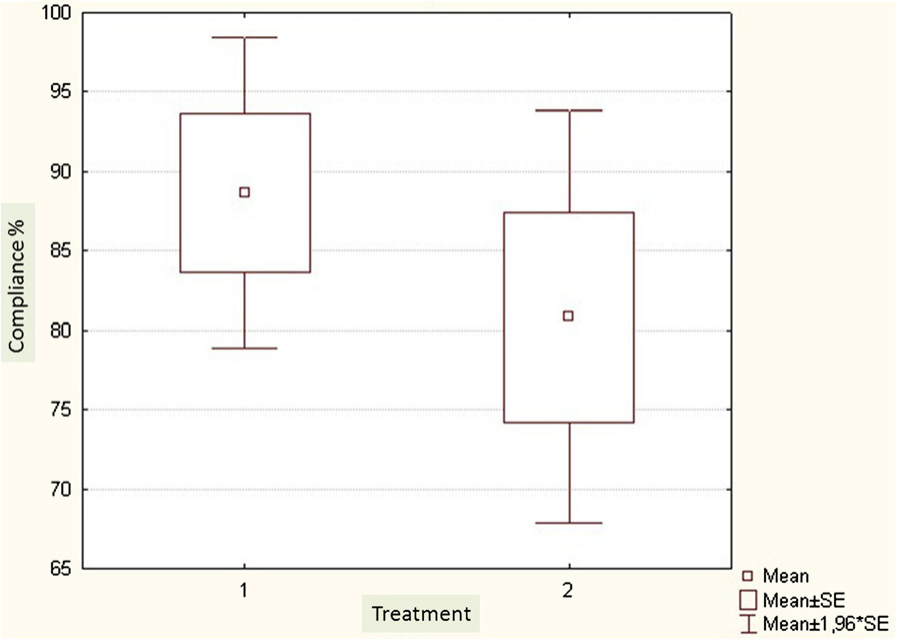
Compliance measured by patients’ own statement. 1: Outpatient group and 2: hospitalized group.

**Figure 5:**
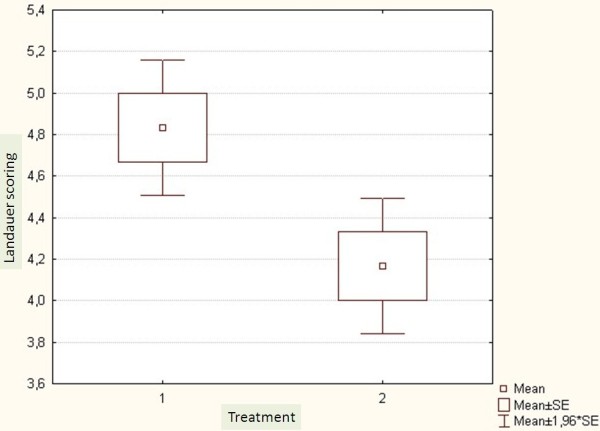
Landauer Compliance scoring. 1: ambulatory group and 2: hospitalized group.

There was no significant difference between the two groups regarding the compliance reported by patients’ own statement (p <0.34) (Figure 4). Landauer Compliance Scoring was better for Op than for the Hp (p < 0.001) (Figure 5).

**Table 2:** Shows the demographics with statistical analysis

	**Op group**	**Hp group**	**P value**
**Number**	**12**	**12**	**NA**
**Age at treatment**	**13 ±1.7**	**12.5 ±2.4**	**0.25**
**Gender**	**Females**	**Females**	**NA**
**Risser grade**	**1.5 ± 1.3**	**1.3 ± 1.2**	**0.43**
**Curve Magnitude**	**36° ±8.6**	**34° ±9.9**	**0.86**
**Curve Type**	**T7,TL3,L2**	**T9,TL2,L1**	**NA**

## Discussion

The effect of brace treatment of AIS is still controversial [5,11,14-16]. The psychosocial effect of brace treatment is well documented though, especially at the initiation phase of treatment [17,18]. A search for alternatives more acceptable for adolescents has included the development of night time only bracing, which minimize the psychological stress and physical discomfort associated with the full time brace treatment [17,18].

Results of brace treatment are difficult to measure because evaluation is multi-factorial. It is well known that the effectiveness of brace treatment depends on the primary in-brace correction and patient compliance [5,11,19]. Compliance is defined as the degree to which patients correctly follow the instructions regarding the brace treatment, and non compliance with brace treatment is a well known problem [11,15]. This is assumed to be worse with the use of full-time braces [6], but studies on this issue are controversial although they do indicate that part-time bracing is better tolerated [3,19].

Hospitalization for brace adaptation and fitting has been practiced for many years. This continues to be the treatment protocols for many centers with the hope of better compliance and better patient satisfaction. The practice differs from one centre to another, but generally admission for two to three days is the norm. In practice, this would mean that the patient and one of the parents should be hospitalized for this period and this could add even more stress on the patients and their families. In this study, we examined whether hospitalization at the initiation phase of brace treatment affects patient satisfaction or compliance. This was evaluated using a validated instrument namely the SQLI and a non-validated OUH questionnaire. This study should be interpreted with the scope of being a prospective consecutive cohort study of two groups. The two groups were matched regarding the age, sex, number, Risser grade, primary correction and severity of scoliosis measured by Cobb angle. Pressure measuring using the Fuji film strip, for the compliance has not been used in this study. Nevertheless, Hasler et al [20], have shown in their study, that the use of pressure strip to measure the compliance could give some bias, as the patients could use the braces more than intended.

To the best of our knowledge, this is the first study that investigates the significance of an outpatient procedure in contrast to hospitalization in the initiation phase of AIS bracing. We found a significant higher patient compliance among the non-hospitalized patients. One could contemplate over the reason for this result. One explanation could be the fact that patients are self dependant, when initiating the treatment at home. Giving this group of patients the feeling that they bear the responsibility for the treatment could have influenced their perception of the importance of treatment. The economical aspects should also be considered, as hospitalization means one or both parents should have free from work.

The weaknesses of the study comprise the rather low number of participants and the short follow-up time. The aim of our study was entirely to evaluate the initial stage of the treatment, and this is not affected by the time of follow-up. This can be explained by the fact that the patient’s own perception of the treatment quality can be reported readily. Indeed, we believe that longer follow-up time could result in bias, as the patient’s satisfaction after a longer follow-up time would depend on the result of the treatment and not the way the treatment was initiated. We believe that compliance is patient, family and physician dependant and cannot be improved by hospital admission.

## Conclusion

Outpatient initiation and fitting of bracing in scoliosis seems to give the same primary correction and patient satisfaction, but gives better compliance compared to initiation during hospitalization. This study suggests that bracing in AIS could be initiated safely as an outpatient procedure, without concerns that this would have negative impact on the patient satisfaction and compliance in the initiation phase.

## Consent

Written informed consent was obtained from the patient for publication of this report and any accompanying images.

## Competing interests

The authors declare that they have no competing interests.

## Authors’ contributions

ZA the corresponding author carried out collection of the data, measuring the X rays, analyzing the data, writing the manuscript. HT helped in analyzing the data. NWP helped out in writing the manuscript. SJ helped out in reading and gave critique of the manuscript. All authors read and approved the final manuscript.
